# Cluster randomized trial of a team communication training implementation strategy for depression screening in a pediatric healthcare system: a study protocol

**DOI:** 10.1186/s43058-024-00641-5

**Published:** 2024-10-18

**Authors:** Nicole A. Stadnick, Gregory A. Aarons, Hannah N. Edwards, Amy W. Bryl, Cynthia L. Kuelbs, Jonathan L. Helm, Lauren Brookman-Frazee

**Affiliations:** 1 Department of Psychiatry, University of California San Diego, La Jolla, CA USA; 2 University of California San Diego Altman Clinical and Translational Research Center Dissemination and Implementation Science Center, La Jolla, CA USA; 3 Child and Adolescent Services Research Center, San Diego, CA USA; 4 Department of Pediatrics, University of California San Diego, La Jolla, CA USA; 5 Rady Children’s Hospital, San Diego, CA USA; 6 Department of Psychology, San Diego State University, San Diego, CA USA; 7 Implementation Science and Team Effectiveness in Practice Children’s Mental Health Research Center, San Diego, USA

**Keywords:** Depression screening, Team communication, Team effectiveness research, Implementation science

## Abstract

**Background:**

Pediatric depression is a global concern that has fueled efforts for enhanced detection and treatment engagement. As one example, the US Preventive Services Task Force recommends depression screening for adolescents ages 12–18 years. While many health systems have implemented components of depression screening protocols, there is limited evidence of effective follow-up for pediatric depression. A key barrier is timely team communication and coordination across clinicians and staff within and across service areas for prompt service linkage. However, team effectiveness interventions have been shown to improve team processes and outcomes and can be applied in healthcare settings.

**Methods:**

This project aims to refine and test a team communication training implementation strategy to improve implementation of an existing pediatric depression screening protocol in a large pediatric healthcare system. The team will be defined as part of the study but is expected to include medical assistants, nurses, physicians, and behavioral health clinicians within and across departments. The implementation strategy will target team mechanisms at the team-level (i.e., intra-organizational alignment and implementation climate) and team member-level (i.e., communication, coordination, psychological safety, and shared cognition). First, the project will use mixed methods to refine the team training strategy to fit the organizational context and workflows. Next, a hybrid type 3 implementation-effectiveness pilot trial will assess the initial effectiveness of the team communication training (implementation strategy) paired with the current universal depression screening protocol (clinical intervention) on implementation outcomes (i.e., feasibility, acceptability, appropriateness, workflow efficiency) and clinical/services outcomes (increased frequency of needed screening and reduced time to service linkage). Finally, the study will assess mechanisms at the team and team member levels that may affect implementation outcomes.

**Discussion:**

Team communication training is hypothesized to lead to improved, efficient, and effective decision-making to increase the compliance with depression screening and timely service linkage. Findings are expected to yield better understanding and examples of how to optimize team communication to improve efficiency and effectiveness in the pediatric depression screening-to-treatment cascade. This should also culminate in improved implementation outcomes including patient engagement critical to address the youth mental health crisis.

**Trial registration:**

NCT06527196.

Trial Sponsor: University of California San Diego.

**Supplementary Information:**

The online version contains supplementary material available at 10.1186/s43058-024-00641-5.

Contributions to the literature

- Effective youth depression screening and care linkage is hindered by implementation challenges including communication and coordination gaps across service systems, organizations, and providers.
- This study will leverage team effectiveness interventions and implementation science by testing a multilevel team communication implementation strategy that targets provider and organizational-level mechanisms to improve the youth depression care cascade.
- Results of this study are expected to inform implementation strategies to enhance timely and more responsive youth depression screening and treatment, which will in turn improve outcomes for pediatric patients in need of mental health care.

## Background

### Depression screening for pediatric patients

Data from the National Survey of Children’s Health indicates a 3.2% prevalence rate for pediatric depression in the US and 14.3% among youths with serious co-occurring and life-limiting medical conditions [1]. Even more concerning are results from a recent meta-analysis indicating a global 25.2% prevalence rate of pediatric depression during COVID-19 [2]. This, along with the recent US Surgeon General’s Advisory on the youth mental health crises that has been exacerbated by the COVID-19 Pandemic, refocuses the need for effective and efficient screening and treatment for pediatric depression [3, 4]. While many pediatric healthcare systems and clinics are aware of this need, there is little evidence of widespread effective follow-up for pediatric depression [5].

### The clinical intervention: depression screening and referral to treatment

The US Preventive Services Task Force recommends depression screening for adolescents 12–18 years old [6]. Many pediatric healthcare systems are implementing depression screening protocols across medical subspecialities, including Rady Children’s Health Network [7]. The screening to treatment “cascade” includes screening, clinical recognition of a problem, referral, treatment initiation, and treatment response [8]. Successful implementation requires effective and optimized inter-provider communication and coordinated treatment planning to facilitate timely review and health system response for at-risk youth.

The implementation context is Rady Children’s Health Network that currently employs a multi-step early identification universal depression screening protocol (the clinical intervention) embedded within the electronic health record (EHR) in multiple specialty units with an existing behavioral health referral service described in Crandal et al. [7]. In this study, all patients who are 12 years and older are expected to be screened using the Patient Health Questionnaire (PHQ) at every encounter within the Emergency Department, primary care, urgent and acute care, inpatient specialty, or within 30 days of outpatient medical appointments if it has not already occurred. Patients are first administered the PHQ-2 [9]. Those who endorse a PHQ-2 score of 3 or greater are then administered the remaining questions on the PHQ-9 [10]. Patients who endorse a PHQ-9 score of 10–19 indicating moderate-severe depression symptomology, but not elevated risk for suicide, are referred to meet with a behavioral health professional and offered educational materials, as well as additional service referrals. This approach focuses primarily on the recognition/identification component of the depression treatment cascade outlined by Pence et al. in 2012 [8].

### Implementation gaps in depression screening

Effective implementation requires a team of individuals across the cascade to communicate and coordinate in a clear and timely manner. Although system-wide interventions such as universal screening policies and mutual access to EHRs between provider specialties can be facilitative, implementation gaps remain in the depression treatment cascade [11]. For example, not all children are screened, and for those who are screened, timely follow-up and linkage to care in response to elevated scores does not consistently occur. More recent and detailed local data [7] support these implementation gaps. All adolescents screened at risk for moderate-severe depression receive a list of behavioral health self-referral options including internal and external services. However, only internal behavioral health referrals are tracked uniformly at Rady Children’s Health Network.

This project proposes that intra-organizational alignment can serve as a mechanism potentially mediating the association of organizational context (e.g., implementation climate) with implementation outcomes [12–14] (e.g., acceptability, appropriateness) to mitigate gaps in the referral to treatment cascade. This approach is consistent with recent developments in organizational research that consider implementation climate and climate strength, a measure of alignment within groups [15]. However, this study will consider intra-organizational alignment across organizational levels. Integrating team effectiveness approaches with implementation science, such as through coordinated specialty care or collaborative care models, is a promising solution to address gaps related to continuous provider communication and coordinated decision-making about evidence-based depression screening, assessment, and service engagement [16–18].

### Team communication training

**Fig. 1:**
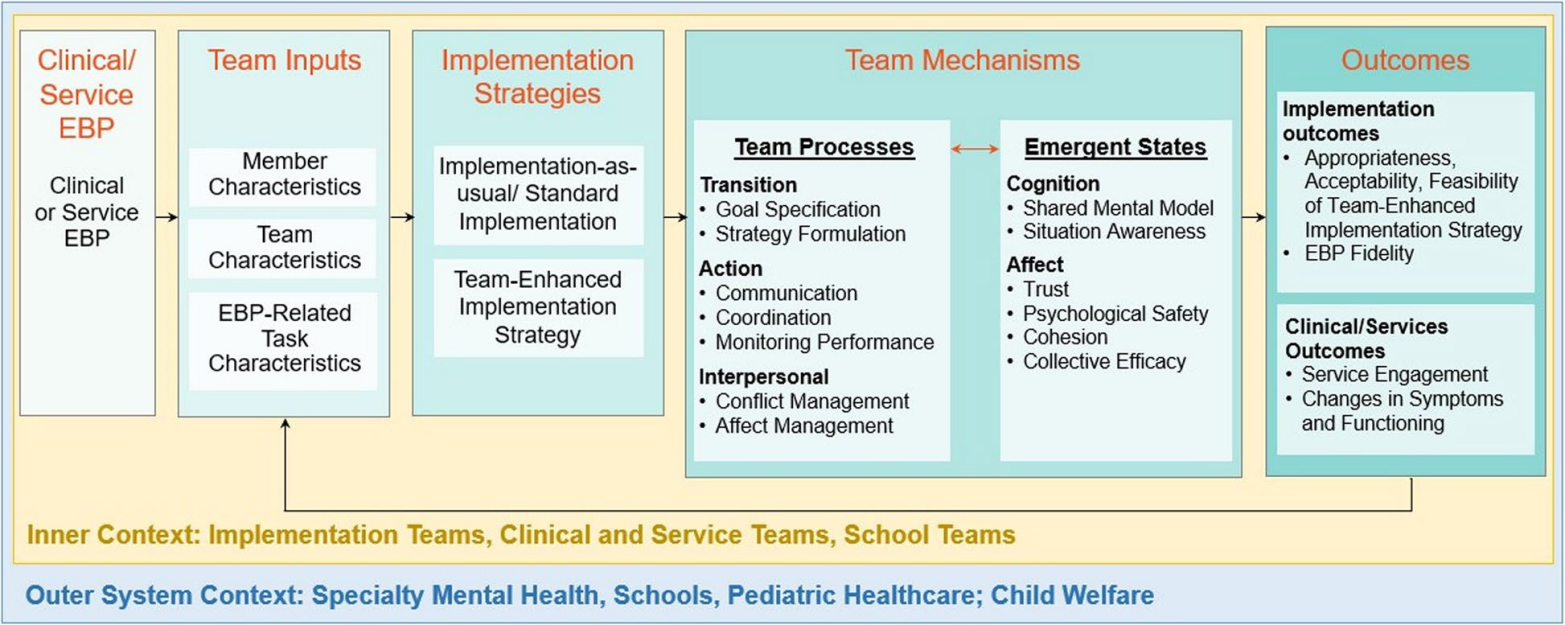
Conceptual model of team effectiveness and implementation science

Team communication training (TCT) is a strategy with a robust evidence base in healthcare settings that has been shown to improve collaborative decision-making, task completion, and health outcomes [19–21]. This study will refine and test TCT (implementation strategy) to target team mechanisms at the team/organizational-level (intra-organizational alignment and implementation climate) and team member/provider-level (communication, coordination, psychological safety, and shared cognition) that will lead to enhanced implementation of an existing pediatric depression screening protocol (clinical intervention). We selected TCT as the team-based implementation strategy because it teaches team members to clearly, concisely, and meaningfully exchange timely and relevant information (i.e., about interpreting depression scores and determining clinical disposition plans). Figure 1 illustrates how this study applies the Implementation Science and Team Effectiveness in Practice (IN STEP) Children’s Mental Health Research Center’s Team Effectiveness for Implementation model [22].

This study will enhance the current active control implementation strategy (automated workflow, decision support tools, and a multidisciplinary taskforce to monitor depression screening compliance and timelines for contacting families) with TCT to improve team mechanisms at the team-level (intra-organizational alignment, implementation climate) and team member-level (shared mental models about roles within the screening/treatment cascade and psychological safety, communication, and coordination within the team). The TCT implementation strategy will be tested using a 2-arm hybrid type 3 pilot trial. We hypothesize that the TCT implementation strategy will engage team mechanisms, which will in turn increase the implementation outcomes of compliance with depression screening and reduced time to service linkage for youth with identified depression risk.

### Assessing team mechanisms at the team and team member levels

Although the team will be refined during the Aim 1 activities, it is expected that the team composition will include medical assistants, nurses, physicians, and behavioral health clinicians (e.g., social workers) within and across departments. Team interactions during a performance cycle impact the formation and refinement of team mechanisms at the team/organizational-level and team member/provider-levels [23]. For example, cognitive emergent states (also known as shared mental models) are important because they are a primary driver of a team’s ability to coordinate interdependent actions [23]. Shared mental models refer to members having a shared understanding of elements such as event milestones, resources, member roles/competencies that are relevant to achieving a team task including effective depression screening, referral, and treatment engagement [23].

In this study, shared mental models refer to shared understanding of the requirements for depression screening, resources following an elevated screen, and role of different team members in the screening to treatment pathway. Also important are affect-based emergent states that drive motivation, effort, and open information exchange as exemplified by psychological safety [23]. The elements of shared cognition can be cognitive and/or affective and indicate the content of shared mental models that can impact team process and outcomes. Improvement in these mechanisms has the potential to improve workflow efficiency by improving “hand-offs” between screening providers and behavioral health assessment/treatment providers. Such hand-offs are associated with increased receipt of services following referral, fewer no-shows or same-day cancellations, fewer days from referral to follow-up behavioral health appointments [24, 25].

We hypothesize that the TCT will be associated with improved implementation outcomes (feasibility, acceptability, adoption, workflow efficiency) and clinical/services outcomes (increased frequency of needed screening and time to service linkage and time to service linkage) compared to the existing universal depression screening implementation condition.

## Method

This study is part of a National Institute of Mental Health Advanced Laboratories for Accelerating the Reach and Impact of Treatments for Youth and Adults with Mental Illness Center [P50MH126231] that supports projects that test team-based implementation strategies that enhance services for children with mental health and developmental needs across systems (schools, specialty mental health, etc.). The project will use mixed methods to refine the team training strategy to fit the organizational context and workflows (Aim 1). A hybrid type 3 implementation-effectiveness pilot trial will then be used to assess the initial effectiveness of the TCT (implementation strategy) paired with the current universal depression screening protocol (clinical intervention) on implementation outcomes (feasibility, acceptability, appropriateness, workflow efficiency) and clinical/services outcomes (increased frequency of needed screening and reduced time to service linkage) (Aim 2). The study will also assess target engagement of mechanisms at the team and team member levels (Aim 3).

### Study objectives

#### Specific Aim 1

Mixed methods will be used to refine a TCT implementation strategy to improve implementation of an existing health system universal depression screening protocol (clinical intervention). We will incorporate organizational team effectiveness research to address implementation challenges associated with health system-wide depression screening protocols. TCT is an effective strategy to enhance communication and decision-making within teams, including in acute healthcare contexts [19–21, 26].

Five focus groups will be conducted with 25 individuals (5 medical specialty clinic leaders, 10 physician or nursing providers, and 10 medical assistants) to identify team (organizational-level) and team member (provider-level) barriers to implementing the existing depression screening protocol. We will also assess workflow efficiency using clinical ethnography and workflow analysis methods [27] to identify specific “pain points” related to screening frequency and time to service linkage. Following rapid analysis [28] of focus group and workflow assessments [27, 29, 30], we will convene a community service working group of pediatric medical specialty clinic and behavioral health care team members to operationalize the components of TCT and tailor for the purposes of increasing the frequency of depression screening and decrease the time to service linkage.

#### Specific Aim 2

A two-arm hybrid type 3 implementation-effectiveness pilot trial will be used to assess the initial effectiveness of the TCT strategy on implementation of the depression screening protocol. The proposed structure and timeline of the TCT is outlined in Table 1. These proposed training components will be refined and finalized based on learnings from the Aim 1 activities with clinic leaders, clinicians, and medical assistants.

**Table 1:** Structure and timeline of team communication training

**Team communication training component**	Month 1				Month 2				Month 3				Month 4				Month 5				Month 6			
	1	2	3	4	5	6	7	8	9	10	11	12	13	14	15	16	17	18	19	20	21	22	23	24
Initial training sessions with didactics and simulation exercises (4 h over 2 sessions)	X		X																					
Team performance feedback about intra-team communication in the EHR provided during regular team meetings (15–20 min)		X		X		X		X		X		X		X		X		X		X		X		X
Booster Coaching Sessions (1 h)								X								X								X

Aim 2 will utilize a cluster randomized design in which four specialty care clinics (10 clinicians per clinic; *n* = 40 clinicians) responsible for reviewing and responding to depression screening (youth ages 12–17 years) will be matched and then randomized to a TCT condition (two clinics) versus an early identification universal depression screening condition (two clinics). Clinics will be matched on team size, makeup of the team members, type of medical care focus and then randomized to conditions. Thus, there will be two sets of two matched clinics that will then be randomized, one to the TCT and the other to early identification universal depression screening condition. In the comparison implementation strategy (early identification universal depression screening), multiple discrete strategies will be used including depression screening training in which providers and staff are provided 1) training in the depression screening tool (PHQ), 2) orientation to the clinical pathway (i.e., screening conducted at every urgent care or emergency department visit and every 30 days for all other medical visits), and 3) expectations for handoff to the next step in the care cascade.

The TCT will last 6 months and is proposed to include the following components that will be refined based on Aim 1 findings and developmental activities: Two initial 2-h didactic sessions on effective communication between team members (information-based methods) and simulation exercises based on mock clinical cases (practice-based methods), biweekly performance feedback about intra-team communication in the EHR (demonstration-based methods) provided during regularly scheduled supervision meetings, and a 1 h booster coaching sessions approximately every 2 months over a 6-month period.

#### Specific Aim 3

Mixed quantitative and qualitative methods will be integrated and used to assess team (intra-organizational alignment, implementation climate) and team member (communication, coordination, psychological safety, shared cognitions) mechanisms of the TCT implementation strategy and a novel application of natural language processing methods. This project will both benefit from and contribute to the IN STEP Methods Core work on Natural Language Processing (NLP). Much of the previous work on team communication relied on hand coding utterances to map them to phases and processes of team cognition [31]. We will explore mechanisms of the TCT implementation strategy through semi-structured interviews and surveys. We will also identify and leverage team communication language data sources that are feasible, naturally occurring, and appropriate for the pediatric health system service context (e.g., transcripts from TCT sessions) to submit to the Methods Core for aggregation across projects using natural language processing methods such as automated utterance coding.

### Setting and context

Participants will be drawn from Rady Children’s Health Network, a comprehensive pediatric healthcare system serving more than 700,000 youth in San Diego County (5th largest county in the U.S.), Imperial, and Riverside Counties [32]. Between 2016 and 2020, 95,613 adolescents were screened using the Rady Children’s Health Network depression screening protocol across outpatient specialty care, the emergency department, inpatient medical units, primary care, and urgent care [7]. Of these, 2.4% screened positive for risk for moderate-severe depression. In the calendar year 2021, 77,425 unique adolescent patients were screened and 9.9% evidenced risk for moderate-severe depression on the PHQ [7].

### Participants

Participants from Aim 1 will be recruited from the outpatient ambulatory departments with the highest rates of depression screening and/or behavioral health referrals based on the most recent patient reports from the EHR. These participants will include clinic leaders who oversee clinical operations including depression screening, clinicians and bedside staff who initiate depression screening such as physicians, nurses, and medical assistants, as well as clinicians who manage triage and referral to behavioral health services including social workers, psychologists, and administrative staff (e.g., patient access representatives).

Participants for Aims 2 and 3 will include 40 clinicians or clinical staff (including physicians, nurses, medical assistants, and mental health providers) drawn from the 4 enrolled specialty care clinics (an average of 10 clinicians per clinic). The specialty care clinic structure comprises all relevant members of the care cascade including a clinician who initiates the depression screening, medical provider who support screening completion, a behavioral health provider who responds to both an elevated depression screen and triages to the patient access representatives who are expected to confirm linkage to a behavioral health provider. Based on data from FY 2023, both inpatient and outpatient specialty services have high rates of depression screening.

Although youth are not participants in this project, the focal pediatric population will be youth ages 12–17 years who have a service encounter in the past year. This age range was selected because it aligns with the age range of the current depression screening protocol at Rady Children’s Health Network [7]. De-identified youth patient data will be abstracted from the EHR to determine movement through the screening-to-treatment cascade and workflow efficiency (i.e., time to complete each step in the cascade and referral quality).

### Measures

Multiple measures will be used to assess the outcomes of TCT for Aims 2 and 3. Please refer to Table 2 for a full list of study constructs, outcomes, and the corresponding measures.

**Table 2:** Study Constructs and measures (Aims 2 and 3)

Construct		Purpose	Source	Measure	Timepoint
Implementation outcomes	Acceptability, Appropriateness, Feasibility	Outcome	Provider	-Acceptability of Intervention Measure -Intervention Appropriateness Measure -Feasibility of Intervention Measure; Qualitative Interviews	6 mos
	Workflow Efficiency	Outcome	EHR and Provider	-Time from screening to referral via EHR timestamps (referral processing) -Referral to appropriate service (referral quality)	
Team-Level mechanisms	Implementation Climate	Mechanism	Team	Jacobs et al. Implementation Climate Measure [33]	BL, 3 mos., 6 mos
	Intra-Organizational Alignment	Mechanism	Team	Alignment is assessed by the degree to which team members from different organizational levels (e.g., physician, nurse, medical assistant, social worker) exhibit similarity on Psychological Safety Climate and Implementation Climate measures	
Team member-level mechanisms	Communication and Coordination	Mechanisms	Team	Collaboration and Satisfaction About Care Decisions (CSACD)—Collaboration	
	Shared Cognitions	Mechanism	Team	Shared Mental Model via card sorting task	
	Psych Safety	Mechanism	Team	Edmondson’s Psychological Safety Climate	
	Team Indicators	Exploratory	Team	NLP data sources	Monthly; 6 mos
Clinical/service outcomes	Child demographics	Sample characteristics	EHR	Family Demographics Questionnaire	BL, 3 mos., 6 mos
	Clinical outcomes	Outcome	EHR	-Response to elevated depression score -Time (days) to service linkage from elevated depression score	BL, 3 mos., 6 mos
	*Mental Health Service Linkage*	*Outcome*	*Caregiver*	*-Report of linkage to any mental health service (internal/Rady and external/outside of Rady) following elevated depression score*	*3 mos., 6 mos*
Setting factors	Medical clinic characteristics		EHR and Provider	-# providers -Average patient volume -Patient case-mix: child race/ethnicity, health insurance (public versus private), age	BL

### Implementation outcomes

#### Acceptability, appropriateness, feasibility of intervention measures

This 12-item instrument will be used to capture perceptions of the acceptability, appropriateness, and feasibility of the TCT strategy [34]. Acceptability refers to the degree to which the intervention (in this case TCT) is appreciated or liked, while appropriateness refers to the intervention’s alignment with the specified setting and stakeholders. Feasibility refers to practicality and the likelihood an intervention can be carried out effectively in its assigned setting. Participants provide a value ranging from 1 (completely disagree) to 5 (completely agree) for each item. An individual subscale for each of the three outcomes (acceptability, appropriateness, and feasibility) can be obtained through averaging response values for each question. Participants will complete this measure at the 6-month timepoint following completion of the Aim 2 trial.

#### Workflow efficiency

Workflow efficiency is a primary outcome for the Aim 2 trial that will be assessed through two different measures. The first, referral processing, will be obtained by capturing the time from screening to referral via EHR (Epic) timestamps. The second, referral quality, will be based on provider reports of referral to appropriate service following depressions screening. Both measures will be collected at the 6-month timepoint following completion of the Aim 2 trial.

### Team/organizational-level mechanisms

#### Implementation climate measure

Implementation climate will be measured with the Jacobs et al. Implementation Climate Measure [33]. Implementation climate was originally defined by Klein and Sorra (1996) as ‘shared summary perceptions of the extent to which their use of a specific innovation is rewarded, supported and expected within their organization’ [35]. To help researchers quantify the concept, Jacobs et. al. developed a 6-item instrument with two items per construct organized by climate dimension and indicating the degree to which the innovation being implemented is expected, supported, and rewarded in the organization and assessed with a Likert-scale [33]. This measure will be administered to Aim 2 participants at baseline, 3 months, and 6 months.

#### Psychological safety

Psychological safety, the degree to which a team member feels it is safe to take interpersonal risks within the work environment, will be measured using Edmonson’s Psychological Safety Climate [36]. This 7-item measure asks participants to rate responses to each statement on a scale of 0 (doesn’t apply at all) to 4 (entirely applies). Scores are subsequently calculated by averaging item responses. The Psychological Safety Climate Measure will be administered to clinical team members at baseline, 3 months, and 6 months.

#### Intra-organizational alignment

Alignment [12, 15] will be measured based on the degree to which team members from different organizational levels (e.g., physicians, nurses, medical assistants, social workers) exhibit similarity on Edmonson’s Psychological Safety climate [36] and the Jacob’s et al. Implementation Climate Measure [33].

### Team member mechanisms

#### Collaboration and satisfaction about care decisions

The Collaboration and Satisfaction about Care Decisions (CSACD) includes 6 items that measure essential components of collaboration [37]. The instrument was originally created to measure nurse-physician interactions in an intensive care unit, and each item captures a specific aspect of collaboration. Values for each question range from 1 (strongly disagree) to 7 (strongly agree) based on a Likert-type scale. Subscale scores are derived by averaging response values for collaboration (3 questions) and satisfaction (3 questions). The CASCD will be administered to participants at baseline, 3 months, and 6 months to gain insight into communication and coordination as it relates to the depression screening treatment cascade.

#### Card-sorting task (shared mental model)

Card sorting is a research assessment to elicit individual mental models about a participant’s understanding of a situation, event, or process. Within team contexts, card sorting can be used to interrogate the extent to which team members have aligned thinking about key elements of a situation, event or process, i.e., team mental models. Each participant from the Aim 2 trial will complete the card-sorting task following TCT completion to assess team mental models of member roles, responsibilities, and goals across depression care cascade team members.

#### Team indicators

Team communication data (e.g., transcripts from TCT sessions, inter-provider messages in the EHR) will be collected during the Aim 2 trial. Labels from a subset of the communication data will be generalized to the larger set of unlabeled utterances using a combination of techniques including semi-supervised learning, pre-trained natural language embeddings, and transfer learning. Semi-supervised learning propagates small label sets by making assumptions about the underlying data distribution and distance function. “Embeddings” are mechanisms for mapping high-dimensional spaces to low-dimensions while retaining the most effective representations, making it possible to apply machine learning on large inputs by representing them in the form of a sparse vector. Transfer learning uses classifiers trained on a source domain to classify data in a specific domain. The IN STEP Methods Core NLP team will consult on the identification of language data most useful and appropriate for the assessment of potential language indicators of team effectiveness mechanisms and outcomes in depression screening and engagement in treatment.

### Clinical/service outcomes

#### Demographics

Age, gender, race, ethnicity, education level, primary language, and caregiver working status/profession will be abstracted from the EHR of pediatric patients. This will be collected at baseline.

#### Clinical outcomes

Clinical outcome measures will include responses to elevated depression score and time (in days) to service linkage from elevated PHQ score. This will be measured with data from the EHR at baseline, 3 months, and 6 months.

#### Mental health service linkage

This measure will be based on caregiver or patient report of linkage to any mental health service either internal Rady or external/outside of Rady following elevated depression score. Values will be collected at 3 months and 6 months.

### Setting factors

#### Medical clinic characteristics

Medical clinic characteristics recorded for the study will include number of clinicians, average patient volume, patient-case mix (child race/ethnicity), preferred language, health insurance (public vs private), gender/sex, and age. These will be derived from the EHR and team members and recorded at baseline.

### Data management

#### Analytic plan

For Aims 2 and 3, we will use multilevel modeling with random intercepts and slopes to test the preliminary effects of TCT on implementation and service outcomes (Aim 2) and mechanisms (Aim 3). Refer to Table 2 for measures of outcomes and mechanisms. Specifically, outcomes include team member-reported acceptability, appropriateness, feasibility and workflow efficiency, and child service outcomes. Potential mechanisms are the proposed mediators of implementation climate, psychological safety climate, communication/coordination, and shared cognitions (see Table 2). Clinic will be included as a covariate (fixed effect). To test the effects of TCT a Group [current implementation vs. TCT] X Time [Baseline, 3, 6 Months] cross-level interaction will be assessed for each outcome. If a statistically significant (*p* < .05) interaction term is evident, simple slopes analyses will be conducted.

Quantitative data and qualitative data will be first analyzed separately. First, quantitative analyses of mechanisms of the TCT implementation strategy will be analyzed using the analytic approach described in Aim 2. To examine the hypothesized mediated effects of implementation strategy condition on implementation and service outcomes, multilevel path analysis will be conducted. Measures of Implementation Climate, Intra-Organizational Alignment, Communication and Coordination, Shared Cognitions, and Psych Safety will be tested as mediators in respective models. The specific mediated effects within these models will be tested using the bias-corrected bootstrap approach [38]. Qualitative interview data will be analyzed using rapid qualitative methods [28] based on a templated summary, deductive approach focused on specified team mechanisms. Specifically, the research team will code transcripts for changes in proposed mechanisms as explanatory factors in implementation and service outcomes. We will then use a mixed-methods analytic approach to synthesize the qualitative and quantitative data collected to examine convergence (i.e., do the two methods confirm or find similar results), complementarity (i.e., do the two methods provide more depth of understanding of research questions), and expansion (i.e., do the two methods provide insights beyond either method alone).

#### Power analysis

To estimate the power to detect statistically significant effects for the relations specified in the MLM, the power program RMASS was used. This program is specifically designed to calculate power for longitudinal data with attrition when a comparison between groups (e.g., current implementation vs. TCT) is of primary interest. To estimate the power, several assumptions were made: (1) An alpha level of .05 (2) a conservative effect size of *d* = .61 (we consider this conservative because a meta-analysis [39] of teamwork interventions reported a large effect size of .92), (3) standard deviations at each time-point that were increasing in magnitude (baseline: SD = 1.00 with SDs increasing by .5 at each subsequent time-point, (4) an overall attrition rate of 15% was specified based on our current trials, and (5) a stationary autoregressive structure (lag 1) was specified for the variance–covariance matrix of the repeated measures, using an autocorrelation value of .70. Given these assumptions and with a sample of 10 clinicians from 4 clinics (*n* = 20 in each implementation arm), we will have 80% power to detect the predicted effect on outcomes.

#### Dissemination plan

Data will be shared with ClinicalTrials.gov. The research team will also share information with key Rady Children’s Hospital Network partners to help develop the universal depression screening protocol and bring team communication practices to the providers that can benefit from this research. Findings will also be compiled and submitted to peer-reviewed scientific journals for publication. All publications will subsequently be shared with the National Center for Biotechnology Information, PubMed, and the US NIH National Library of Medicine.

## Discussion

Although several healthcare systems have initiated robust depression screening protocols in response to rising depression rates in the pediatric population [5], there is a gap between screening and successful service linkage. Inconsistent communication between providers, particularly across different specialties and service areas, is a key hindrance to an efficient, effective depression treatment cascade. By leveraging team effectiveness research to develop an enhanced TCT strategy, this study can potentially help improve existing depression screening protocols, thereby increasing the frequency of successful mental health service linkage.

Multiple considerations have been made to optimize study operations, but the research team acknowledges certain barriers or limitations that could interfere with a successful workflow. Some organizations and clinicians prefer virtual training that solves issues of travel, time away from clinics, and financial burden. For this reason, TCT will be positioned to be delivered either remotely or in-person.

However, as with any virtual communication, there is always a risk of technology issues that will affect recording focus groups and/or delivering the intervention during Aim 2. These are critical considerations for scalability of strategies such as TCT. In addition, not all staff members may have the adequate time, understanding, or resources to benefit from synchronous virtual training. To mitigate this, medical clinic leaders (physicians and staff) will be engaged during Aim 1 to build buy-in for the TCT activities and to best coordinate research activities within or around clinical operations. The team will also explore approaches such as asynchronous learning that combine recorded and interactive trainings with applied team participation in exercises to consolidate learning and provide teams with experience and practice. Additionally, the research team acknowledges that asking staff members questions during focus groups or qualitative interviews about their experiences with depression screening may give rise to some uncomfortable feelings or reactions. To mitigate this issue, researchers will adhere to Institutional Review Board (IRB) guidelines by providing all participants with an information sheet that reminds them that study participation is voluntary, emphasizing that they can refuse to answer a question or cease participation at any time.

The implementation strategy refined for the depression screening context (Aim 1) and data collected on its effectiveness (Aim 2) and mechanisms (Aim 3) will serve as essential preliminary data for a subsequent definitive test of the TCT implementation strategy. TCT is likely to lead to improved, efficient, and effective decision-making to increase the frequency of depression screening and timely response vis-à-vis appropriate service linkage. We will also identify and leverage team communication language data sources (e.g., focus group transcripts; recordings of team meetings; team meeting notes and written communication) to submit to the IN STEP Methods core for aggregation across IN STEP Center projects. Eventually, measures developed in this process could be used to further develop and guide team training and development activities.

The refined TCT model from this study is expected to result in a better understanding and examples of how to optimize team communication activities and patterns for efficiency and effectiveness in the screening to treatment cascade. This should result in better patient engagement and outcomes. To accomplish and test assumptions, data and results will be used to refine the TCT model and finalize the intervention for a Hybrid Type 3 application focused on scale-up and sustainment of TCT across pediatric health systems implementing depression screening protocols.

## Supplementary Information

Supplementary Material 1.

Supplementary Material 2.

## Data Availability

N/A.

## Abbreviations

EHR: Electronic Health Record
IN STEP: Implementation Science and Team Effectiveness in Practice
NLP: Natural Language Processing
PHQ: Patient Health Questionnaire
TCT: Team Communication Training
