# Supplementary material for: Cluster randomized trial of a team communication training implementation strategy for depression screening in a pediatric healthcare system: a study protocol
Source: Implement Sci Commun. 2024 Oct 18;5:117. doi: 10.1186/s43058-024-00641-5 (PMC11487972; doi:10.1186/s43058-024-00641-5)
Supplement: Supplementary file 1 — Supplementary Material 1. [file 43058_2024_641_MOESM1_ESM.doc]

**
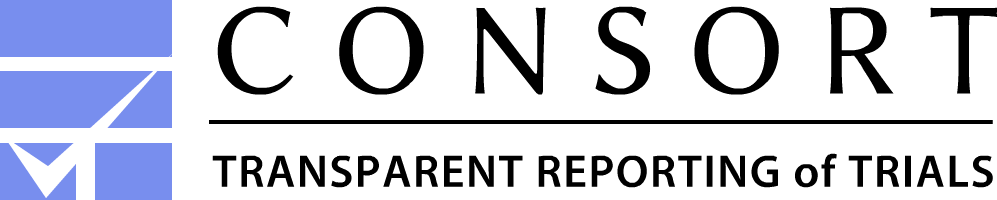
**

**CONSORT 2010 Flow Diagram—Aim 2**

**Allocation & Consent**

**Analysis**

**Post-Intervention Assessment**

**Enrollment & Screening**

Four speciality care clinics divided into two matched sets based on team size, makeup of team members and type of medical care focus

(n=40 clinicians)

Analyzed (n= )
♦ Excluded from analysis (give reasons) (n= )

Completed post-intervention assessment (n= )

Did not complete (n= )

Clinicians allocated to Early Identification Universal Depression Screening (n=20)

♦ 10 clinicians per clinic

♦ Consent to study (n= )

♦ Declined participation (n= )

♦ Declined participation (n= )

Completed post-intervention assessment (n= )

Did not complete (n= )

Clinicians allocated to Team Communication Training (n=20)

♦ 10 clinicians per clinic

♦ Consent to study (n= )

♦ Declined participation (n= )

Analyzed (n= )
♦ Excluded from analysis (give reasons) (n= )

Clinics randomized to condition; not yet recruited (n=40 clinicians)
