# Supplementary material for: Cluster randomized trial of a team communication training implementation strategy for depression screening in a pediatric healthcare system: a study protocol
Source: Implement Sci Commun. 2024 Oct 18;5:117. doi: 10.1186/s43058-024-00641-5 (PMC11487972; doi:10.1186/s43058-024-00641-5)
Supplement: Supplementary file 2 — Supplementary Material 2. [file 43058_2024_641_MOESM2_ESM.doc]

|  |  |  | | | |
| --- | --- | --- | --- | --- | --- |
|  |  | STUDY PERIOD | | | |
|  | Enrollment | Allocation | Start of Intervention | Post-Intervention Assessment | Close-out |
| TIMEPOINT |  |  |  |  |  |
| Enrollment |  |  |  |  |  |
| Eligibility Screen | X |  |  |  |  |
| Informed Consent | X |  |  |  |  |
| Allocation |  | X |  |  |  |
| Interventions |  |  |  |  |  |
| Early Identification Universal Depression Screening |  |  |  |  |  |
| Team Communication Training (TCT) |  |  |  |  |  |
| Assessments: |  |  |  |  |  |
| Alignment |  |  | X | X |  |
| Acceptability of Intervention* |  |  |  | X |  |
| Card-Sorting Task (Shared Mental Model) |  |  | X | X |  |
| Collaboration & Satisfaction About Care Decisions (CSACD)—Collaboration |  |  | X | X |  |
| Demographics |  |  | X | X |  |
| Edmonson’s Psychological Safety Climate Measure |  |  | X | X |  |
| Feasibility of Intervention Measure (FIM)* |  |  |  | X |  |
| Intervention Appropriateness Measure (IAM)* |  |  |  | X |  |
| Jacobs et. al Implementation Climate Measure |  |  | X | X |  |
| Mental Health Service Linkage |  |  |  | X |  |
| Time to Mental Health Service Linkage |  |  | X | X |  |
| Workflow Efficiency (Referral Processing Time) |  |  |  | X |  |
| Workflow Efficiency (Referral Quality) |  |  |  | X |  |

*Assessment only administered to TCT intervention condition
